# Irregular Meal Timing Is Associated with *Helicobacter pylori* Infection and Gastritis

**DOI:** 10.5402/2013/714970

**Published:** 2012-12-30

**Authors:** Su-Lin Lim, Claudia Canavarro, Min-Htet Zaw, Feng Zhu, Wai-Chiong Loke, Yiong-Huak Chan, Khay-Guan Yeoh

**Affiliations:** ^1^Dietetics Department, National University Hospital, 5 Lower Kent Ridge Road, Main Building, Level 1, Singapore 119074; ^2^Dietetics and Nutrition Department, Alexandra Hospital, Jurong Health, Level 1, 378 Alexandra Road, Singapore 159964; ^3^Research and Strategic Planning Division, Research and Evaluation Department, Health Promotion Board, 3 Second Hospital Avenue, Singapore 168937; ^4^Department of Gastroenterology and Hepatology, National University Hospital, 5 Lower Kent Ridge Road Tower Block, Level 10, Singapore 119074; ^5^Global Healthcare Practice, KPMG, 16 Raffles Quay No. 22-00, Hong Leong Building, Singapore 048581; ^6^Biostatistics Unit, Clinical Research Centre, Yong Loo Lin School of Medicine, National University of Singapore, Block MD 11, Level 1, Singapore 117597

## Abstract

*Helicobacter pylori* (HP) is associated with chronic gastritis and gastric cancer, and more than half of the world's population is chronically infected. The aim of this retrospective study was to investigate whether an irregular meal pattern is associated with increased risk of gastritis and HP infection. The study involved 323 subjects, divided into three groups as follows: subjects with HP infection and gastritis, subjects with gastritis, and a control group. Subjects were interviewed on eating habits and meal timing. Multivariate logistic regression was used to compare groups. Adjusted odds ratios (OR) were derived controlling for gender, age, stress, and probiotic consumption. Subjects who deviated from their regular meals by 2 hours or more had a significantly higher incidence of HP infection with gastritis (adjusted OR = 13.3; 95% CI 5.3–33.3; *P* < 0.001) and gastritis (adjusted OR = 6.1; 95% CI 2.5–15.0; *P* < 0.001). Subjects who deviated their meals by 2 hours or more, twice or more per week, had an adjusted OR of 6.3 and 3.5 of acquiring HP infection with gastritis (95% CI 2.6–15.2; *P* < 0.001) and gastritis (95% CI 1.5–8.5; *P* < 0.001), respectively. Frequent deviation in meal timing over a prolonged period appears associated with increased risk of developing HP infection and gastritis.

## 1. Introduction

Since the discovery of *Helicobacter pylori* (HP) in the 1980s, considerable attention has been given to this bacterium as a cause of gastritis and an established risk factor for gastric cancer [1–3]. *Helicobacter pylori* is known to chronically infect more than half of the world's population [4]. Infection is common in Singapore, affecting 71% of adults above 65 years and 3% of children below 5 years [5]. 

*Helicobacter pylori* infection is associated with a complex interaction between genetic [6], socioeconomic [7], environmental [8], and bacterial factors [9]. This results in multiple potential outcomes following infection, including chronic gastritis and gastric adenocarcinoma [10, 11]. Due to the close association between HP, gastritis and gastric cancer, it is of interest to decrease the occurrence of HP infection and gastritis.

To date, there is a scarcity of published literature on the impact of irregular meals on HP infection or gastritis. This study aims to determine whether a prolonged irregular meal pattern is associated with increased risk of gastritis and HP infection.

## 2. Materials and Methods

### 2.1. Ethics

The study protocol was approved by the National Healthcare Group Domain Specific Review Board. Consent was obtained from participants before the survey was carried out.

### 2.2. Sample-Size Calculation

The sample size was calculated based on a community survey of 113 people in Singapore prior to the commencement of this study, which showed 16% had irregular meals. Postulating that this prevalence would double in subjects with HP and gastritis, 120 subjects per group has a power of 80% and a 2-sided test of 5% to achieve a statistically significant result.

## 3. Subjects

**Figure 1:**
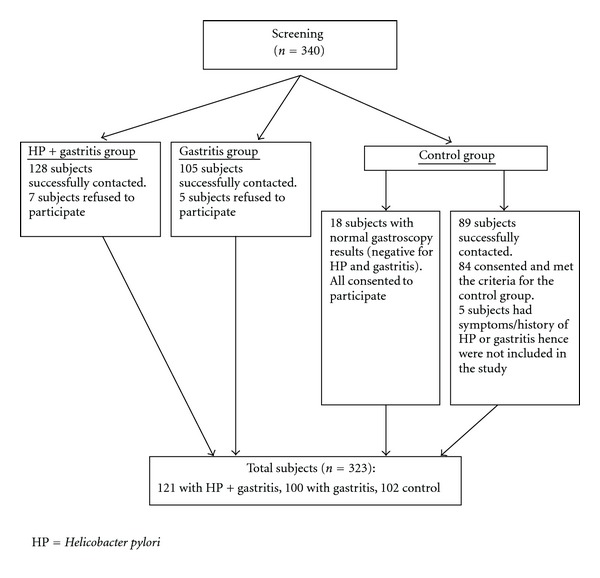
Recruitment process.

All subjects were of Chinese ethnic origin and aged 50 years and above, in order to minimize the confounding factors of age and race. A total of 323 subjects were divided into three groups according to HP and gastritis status. The HP and gastritis group (Group A) consisted of patients diagnosed with HP and gastritis (*n* = 121). The gastritis group (Group B) consisted of patients who had been diagnosed with gastritis but negative to HP (*n* = 100). All patients in Group A and B had undergone endoscopic biopsy, with gastritis and HP diagnosed from mucosal biopsy in three locations (antrum, body, and cardia) and by consensus amongst three pathologists according to the updated Sydney System for the classification and grading of gastritis [12]. Subjects in the control group (group C) had normal endoscopic biopsy results (*n* = 18) or no symptoms or history of gastritis or HP (*n* = 84 community-recruited subjects) (*n* = 102). We compared the diet patterns of the 18 participants with endoscopy results to the 84 without endoscopy and found the patterns were similar (*P* much greater than 0.05 in all parameters). Details of the recruitment process are described in Figure 1.

### 3.1. Data Collection

All subjects were administered a specially designed questionnaire by two trained dietitians. This included questions regarding regularity of meals, the frequency and duration of any changes to usual meal timing, variation in the amount of food eaten, and the practice of skipping meals. Subjects in Groups A and B were surveyed regarding their eating patterns prior to the diagnosis of HP or gastritis. Subjects in the control group (Group C) were asked to respond regarding their eating pattern prior to endoscopy, or prior to interview for the community recruited subjects. 

We defined irregular meals as a deviation from regular meal timing for 1 hour or more at least once per week. Questions regarding the practice of skipping meals were worded to detect subjects who omitted non-corresponding meals of the day (i.e., not the same meal every day). Subjects who missed the same meal each day were considered to have a regular meal pattern consisting of one less meal per day. The questionnaire also surveyed probiotic consumption, the presence of stress or any major stressful event prior to diagnosis, to enable these to be addressed as confounders. 

### 3.2. Statistical Analysis

All analyses were performed using SPSS 17.0 with statistical significance set at *P* < 0.05. Multivariate logistic regression was performed to determine the risk predictors for the HP with gastritis and gastritis groups. Unadjusted odds ratios were derived comparing group A versus control (group C) and group B versus control (group C) using chi-square or Fisher's Exact test. Adjusted odds ratios were derived controlling for gender, age, stress, and consumption of probiotics.

## 4. Results

Table 1 describes the demographics of the study subjects. There were no significant differences in age and gender distribution across the 3 study groups. 

### 4.1. Deviation in Meal Timing

Table 2 shows that the adjusted odds ratio (OR) of developing HP with gastritis (Group A) and gastritis (Group B) increased as the time of meal deviation increased. A deviation in meal timing of equal to or more than 2 hours was associated with a significant risk of developing HP with gastritis or gastritis, with an adjusted OR of 13.3 (95% CI 5.3–33.3, *P* < 0.001) and 6.1 (95% CI 2.5–15, *P* < 0.001), respectively. The adjusted OR for developing HP with gastritis and gastritis also increased as the frequency of meal deviation increased (Table 3). Subjects in Group A who deviated their meals equal to or more than twice per week had an adjusted OR of 4.4 of developing HP infection with gastritis (95% CI  2.3–8.7, *P* < 0.001). Those in Group B had an adjusted OR of 3.8 of developing gastritis (95% CI 1.9–7.6,  *P* < 0.001).

Table 4 shows that subjects who deviated from their regular meals by two or more hours, twice or more per week, were associated with significantly higher incidence of HP infection with gastritis (adjusted OR = 6.3, 95% CI 2.6–15.2, *P* < 0.001) and gastritis (adjusted OR = 3.5, 95% CI 1.5–8.5, *P* < 0.005). There were significant differences in the mean period of meal deviation between the HP with gastritis, gastritis and control groups (7.9 years versus 8.1 years versus 4.5 years, *P* < 0.001) (Table 5).

### 4.2. Skipped Meals

Although the proportion of subjects who skipped meals almost doubled in the HP with gastritis and gastritis groups in comparison to those in the control group (19% versus 9.8%), there was no significant difference between the groups (Table 6).

### 4.3. Inconsistent Amount of Food Consumption

There was no significant difference between groups for subjects who had an inconsistent amount of food at each meal (Table 7). 

## 5. Discussion

This study is the first to examine an association between the degree of irregularity in meal timing and risk of HP and gastritis. After controlling for the potential confounders of gender, age, stress, and consumption of probiotics, we found that deviating from regular meal timing by two hours or more was associated with a thirteenfold increase in risk of developing HP with gastritis, and a sixfold increase in risk of developing gastritis. 

The association of dietary habits with the development of HP infection has been given relatively little attention. A number of studies have demonstrated evidence of an association between intake of specific food or nutrients and HP [13–16]. However fewer studies exist examining the relationship between irregular meals and gastritis, and none have studied the degree of irregularity in meal timing [17, 18]. A retrospective questionnaire study involving 76 men and 19 women with peptic ulcers in Japan found that eating irregular meals significantly increased the relative risk of peptic ulcer in men, but not in women. In this instance the small number of women subjects may not have provided enough power for statistical significance [17]. One Chinese study revealed a significant correlation between irregular meals and gastric cardia cancer with an odds ratio of 4.2 [19]. However in both studies, there was no mention how irregularity in meals was surveyed, and whether deviation in meal timing, omitted meals, and variations in food quantity were included.

Bulgarian researchers who found an increase in radiologically documented gastroduodenal ulcers during a period of economic crisis reported their impression that skipped meals and chain smoking were contributory factors [18]. The role of traditional risk factors on the prevalence of duodenal ulcer disease was investigated at an endoscopy unit in Jordan with high prevalence of HP amongst patients. Skipping breakfast or more than one meal was found to be among important factors in the predisposition for ulcer disease in subjects with HP [20].

In this study, meal regularity and habits prior to the diagnosis of HP infection and gastritis were evaluated. The odds ratio increased as the deviation in meal timing increased in the case groups when compared to the control group. Not everyone who is exposed to HP will become infected [21]. Before a person can be infected with HP, the bacteria must penetrate the gastric mucosa [22]. The gastric mucosa acts as a natural protective barrier, which limits the penetration of microorganisms [23]. However we do not yet know whether irregularity in meal timing changes the mucosal membrane and increases susceptibility to bacterial penetration. It has recently been established that the barrier function of the mucosa can be disturbed under a variety of pathological insults [24]. We hypothesize that people who have irregular meals are at higher risk of HP infection or gastritis because during the usual meal timing the stomach and intestines produce secretions, free radical scavengers or perhaps some other yet to be discovered chemical, in readiness to receive food. If food is not ingested during this time, the secretions or lack of secretions somehow cause the lining of the stomach to be susceptible to HP infection and gastritis. It has been established that HP survives in brief exposure to acidic pH of less than 4 and growth occurs only at the relatively narrow pH range of 5.5 to 8.0, with optimal growth at neutral pH [25, 26]. Upon entry to the host, spiral morphology and flagellar motility facilitates penetration of the more pH neutral viscous mucosal layer for infection to occur [27, 28]. This could be indicative of a possible effect of meal timing deviation on the gastric pH that makes the mucosa susceptible to HP infection. 

The mean duration of meal timing deviation in this study was about 8 years for the case groups compared to 4.5 years in the control group. It has been suggested that to cause harm, HP must efficiently adapt to the gastric niche, a process that takes place over many years and involves regulation of bacterial genes in response to environmental factors [29]. These environmental factors may include, but are not limited to, cigarette smoking, stress, irregularity in meal timing and other dietary factors. Stress has been shown to increase gastric permeability to pathogens such as HP [30, 31].

It is well established that HP infection leads to gastritis [32]. Although our study showed a significant association between irregular meal timing and gastritis as well as occurrence of HP infection, it did not determine whether irregularity in meal timing is the cause or effect of these. We postulate that frequent deviation from regular timing of meals is likely to cause gastritis or HP infection. Glutathione level has been found to be elevated in HP infection and some forms of gastritis [33, 34]. Irregularity in meal timing possibly increases glutathione production in the stomach. In addition it may also cause low gastric acid secretion and studies have shown that clinical conditions with low gastric acid secretion are associated with increased risk of gastric cancer [35, 36]. Meal timing may also impact physiological parameters such as endocrine variables [37]. In our study, the significant differences in the regularity of meal timing of the HP with gastritis and gastritis groups in comparison to the control group supports the presence of the above mechanisms.

The merits of this study are the fairly large sample size and the use of endoscopic biopsy as endpoint for diagnosis in three quarters of the study population. Due to ethical issues, endoscopic biopsies were not carried out on the community-recruited subjects in the control arm. However, comparison of participants with and without endoscopy results showed similar diet patterns and baseline characteristics (analyses not shown in this paper). Some subjects in the control group may have had HP without their knowledge, as individuals may remain asymptomatic despite having HP [38]. Active chronic gastritis may also not present any clinical symptoms [27]. If we had been able to definitively exclude HP or gastritis in these community-recruited subjects it may have further strengthened the results of this study, as 39% of subjects in the control group had irregular meals. 

A major limitation of this study was the retrospective design and its inability to provide causal link of HP infections and gastritis to irregular eating patterns. Surveyor and respondent bias were further limitations in this retrospective study. Significant recall bias was possible with the self-reported questionnaires. An individual with chronic gastritis or HP infection might likely be much more aware of their dietary habits than a healthy control. In addition, the dietitians administering the questionnaire were not blinded to the participants' diagnosis. lt

## 6. Conclusion

In conclusion, a variation in meal timing over a prolonged period appears to be associated with increased risk of symptomatic HP infection and gastritis. Regular timing of meals may play an important role in the prevention of these two medical conditions. As there is a scarcity of published data studying an association between irregular meal timing and HP and gastritis, this pilot paper warrants future prospective studies to determine the effect of irregular meals on the development of gastritis and HP. 

## Figures and Tables

**Table 1:** Demographics of study subjects.

	HP + Gastritis (Group A) (*n* = 121)	Gastritis (Group B) (*n* = 100)	Control (Group C) (*n* = 102)	*P* value
Age (year)				0.239
Mean ± SD	62.5 ± 7.3	63.1 ± 7.5	61.0 ± 7.0	
Range	51–79	52–87	50–82	
Gender				0.163
Male	71 (58.7%)	51 (51.0%)	47 (46.1%)	
Female	50 (41.3%)	49 (49.0%)	55 (53.9%)	

HP: *Helicobacter pylori*.

**Table 2:** Relationship between deviation from regular meals by number of hours and *Helicobacter pylori* infection with gastritis and gastritis.

Deviation from regular meals	Control Count (%)	HP* + *Gastritis (Group A) Count (%)	Gastritis (Group B) Count (%)	Group A versus Control: Odds Ratio (95% Confidence Interval) *P* value		Group B versus Control: Odds Ratio (95% Confidence Interval) *P* value	
				Unadjusted	Adjusted^#^	Unadjusted	Adjusted^#^
0 to <1 hour	62 (60.8%)	38 (31.4%)	28 (28%)	OR = 1.0	OR = 1.0	OR = 1.0	OR = 1.0
≥1 to <1.5 hours	26 (25.5%)	24 (19.83%)	33 (33%)	OR = 1.5 (0.76–3.0) *P* = 0.242	OR = 1.5 (0.74–3.2) *P* = 0.249	OR = 2.8 (1.4–5.6) *P* = 0.003*	OR = 2.5 (1.2–5.0) *P* = 0.014*
≥1.5 to <2 hours	5 (4.9%)	11 (9.09%)	8 (8%)	OR = 3.6 (1.2–11.1) *P* = 0.027*	OR = 4.2 (1.3–14.1) *P* = 0.019*	OR = 3.5 (1.1–11.8) *P* = 0.039*	OR = 3.3 (0.96–11.6) *P* = 0.058
≥2 hours	9 (8.8%)	48 (39.67%)	31 (31%)	OR = 8.7 (3.8–19.7) *P* < 0.001*	OR = 13.3 (5.3–33.3) *P* < 0.001*	OR = 7.6 (3.2–18.1) *P* < 0.001*	OR = 6.1 (2.5–15.0) *P* < 0.001*

HP: *Helicobacter pylori*, OR: Odds ratio.

^#^Adjusted for gender, age, stress, and use of probiotics.

*Statistical significance.

Note: there were no statistical differences in the frequency of deviation from regular meals between groups A and B.

**Table 3:** Relationship between deviation from regular meals by frequency per week and *Helicobacter pylori* infection with gastritis and gastritis.

Frequency of meal deviation per week	Control Count (%)	HP* + *Gastritis (Group A) Count (%)	Gastritis (Group B) Count (%)	Group A versus Control: Odds Ratio (95% Confidence Interval) *P* value		Group B versus Control: Odds Ratio (95% Confidence Interval) *P* value	
				Unadjusted	Adjusted	Unadjusted	Adjusted
0	62 (60.8%)	38 (31.4%)	28 (28%)	OR = 1.0	OR = 1.0	OR = 1.0	OR = 1.0
1	15 (14.7%)	25 (20.66%)	21 (21%)	OR = 2.7 (1.3–5.8) *P* = 0.01*	OR = 2.9 (1.3–6.5) *P* = 0.008*	OR = 3.1 (1.40–6.89) *P* = 0.006*	OR = 2.7 (1.1–6.2) *P* = 0.023*
≥2	25 (24.51%)	58 (47.93%)	51 (51%)	OR = 3.8 (2.0–7.0) *P* < 0.001*	OR = 4.4 (2.3–8.7) *P* < 0.001*	OR = 4.52 (1.1–11.8) *P* < 0.001*	OR = 3.8 (1.9–7.6) *P* < 0.001*

HP: *Helicobacter pylori*, OR: Odds ratio.

^#^Adjusted for gender, age, stress, and use of probiotics.

*Statistical significance.

Note: there were no statistical differences in frequency of meal deviation between groups A and B.

**Table 4:** Distribution and odds ratio for subjects who deviate from regular meals stratified by hours and frequency of meal deviation.

	Subjects who deviated from their regular meals by ≥1 hour for ≥2 times per week			Subjects who deviated from their regular meals by ≥2 hours for ≥2 times per week		
	Count (%)	Unadjusted Odds Ratio (95% CI) *P* value	Adjusted^#^ Odds Ratio (95% CI) *P* value	Count (%)	Unadjusted Odds Ratio (95% CI) *P* value	Adjusted^#^ Odds Ratio (95% CI) *P* value
Control (*n* = 102)	25 (24.5%)	OR = 1	OR = 1.0	8 (7.8%)	OR = 1	OR = 1.0
HP *+ *Gastritis (Group A) (*n* = 121)	58 (47.9%)	OR = 2.8 (1.6–5.0) *P* < 0.001*	OR = 3.1 (1.7–5.8) *P* < 0.001*	34 (28%)	OR = 4.6 (2.0–10.5) *P* < 0.001*	OR = 6.3 (2.6–15.2) *P* < 0.001*
Gastritis (Group B) (*n* = 100)	51 (51%)	OR = 3.2 (1.8–5.8) *P* < 0.001*	OR = 2.9 (1.5–5.3) *P* = 0.001*	26 (26%)	OR = 4.1 (1.8–9.6) *P* = 0.001*	OR = 3.5 (1.5–8.5) *P* = 0.005*

HP: *Helicobacter pylori*, OR: Odds ratio.

^#^Adjusted for gender, age, stress, and use of probiotics.

*Statistical significance.

Note: there were no statistical differences in frequency of meal deviation between groups A and B.

**Table 5:** Mean period of meal deviation habit for *Helicobacter pylori* with gastritis, gastritis, and control group.

	Mean ± SD (years)	95% Confidence Interval for Mean	Difference between groups
Control (*n* = 102)	4.5 ± 6.7	3.2–5.8	*P* < 0.001*
HP + Gastritis (Group A) (*n* = 121)	7.9 ± 7.1	6.6–9.1	
Gastritis (Group B) (*n* = 100)	8.1 ± 7.2	6.7–9.6	
Total (*n* = 323)	6.9 ± 7.2	6.1–7.7	

HP: *Helicobacter pylori*.

*Statistical significance.

**Table 6:** Distribution and odds ratio for subjects who regularly skipped meals.

	Omitted meal at least one time per week Count (%)	Unadjusted Odds ratio (95% CI) *P* value	Adjusted^#^ Odds ratio (95% CI) *P* value
Control (*n* = 102)	10 (9.8%)	OR = 1	OR = 1
HP + Gastritis (Group A) (*n* = 121)	23 (19%)	OR = 2.2 (0.98–4.8) *P* = 0.058	OR = 2.3 (0.99–5.5) *P* = 0.053
Gastritis (Group B) (*n* = 100)	19 (19%)	OR = 2.2 (0.95–4.9) *P* = 0.067	OR = 2.2 (0.9–5.3) *P* = 0.094

HP: *Helicobacter pylori*, OR: Odds ratio.

^#^Adjusted for gender, age, stress, and use of probiotics.

Note: there were no statistical differences in frequency of skipped meals between groups A and B.

**Table 7:** Distribution and odds ratio for subjects who had an inconsistent amount of food for each meal.

	Inconsistent amount of food in corresponding meals Count (%)	Unadjusted Odds ratio (95% CI) *P* value	Adjusted^#^ Odds ratio (95% CI) *P* value
Control (*n* = 102)	9 (8.8%)	OR = 1	OR = 1.0
HP *+ *Gastritis (Group A) (*n* = 121)	18 (14.9%)	OR = 1.8 (0.77–4.2) *P* = 0.172	OR = 1.8 (0.8–4.4) *P* = 0.177
Gastritis (Group B) (*n* = 100)	20 (20%)	OR = 2.6 (1.1–6.0) *P* < 0.027*	OR = 2.1 (0.8–5.3) *P* = 0.095

HP: *Helicobacter pylori*, OR: Odds ratio.

^#^Adjusted for gender, age, stress, and use of probiotics use.

*Statistical significance.

Note: there were no statistical differences in the frequency of inconsistent amount of food between groups A and B.
